# Dynamic thiol-disulfide homeostasis as an oxidative stress marker in ankylosing spondylitis and undifferentiated spondyloarthropathy

**DOI:** 10.55730/1300-0144.5705

**Published:** 2021-04-10

**Authors:** Cemaleddin ÖZTÜRK, Şükran ERTEN, Murat ALIŞIK, İsmail DOĞAN, Güniz Yanık ÜSTÜNER, Salim NEŞELİOĞLU, Özcan EREL

**Affiliations:** 1 Division of Hematology, Department of Internal Medicine, Faculty of Medicine, Ankara University, Ankara, Turkiye; 2 Division of Rheumatology, Department of Internal Medicine, Faculty of Medicine, Ankara Yıldırım Beyazıt University, Ankara, Turkiye; 3 Department of Biochemistry, Faculty of Medicine, Bolu İzzet Baysal University, Bolu, Turkiye; 4 Department of Internal Medicine Department, Faculty of Medicine, Ankara Yıldırım Beyazıt University, Ankara, Turkiye; 5 Department of Biochemistry, Faculty of Medicine, Ankara Yıldırım Beyazıt University, Ankara, Turkiye

**Keywords:** Ankylosing spondylitis, spondyloarthropathy, thiol, disulfide, oxidative stress, tumor necrosis factor-a antagonist

## Abstract

**Background/aim:**

Seronegative spondyloarthropathies (SpA) are a group of chronic diseases characterized by axial inflammation, oligoarthritis, and enthesitis. Oxidative stress may contribute to a wide range of rheumatologic diseases, including SpA. This prospective case-control study was designed to compare thiol-disulfide levels as a marker of oxidative stress between SpA patients and healthy controls.

**Materials and methods:**

A total of 144 patients diagnosed with undifferentiated spondyloarthropathy (USpA, n = 97) or ankylosing spondylitis (AS, n = 47) were included along with 80 healthy controls. Serum native thiol (NT), total thiol (TT), and disulfide (D) levels were measured using the fully automated Erel method. The ratios NT/TT, D/TT, and D/NT were calculated. Thiol-disulfide levels were compared between the SpA groups and the healthy controls.

**Results:**

The NT and NT/TT ratios were found to be significantly lower in the SpA group (p < 0.001). The disulfide, D/NT, and D/TT ratios were found to be significantly higher in the SpA group (p < 0.001). In pairwise comparisons between the SpA subgroups, the NT and TT levels were lower in the USpA group than in the AS group (p = 0.021), but serum disulfide levels were higher in the USpA group than in the AS group (p = 0.004). Among the patients with SpA, the group taking antitumor necrosis factor (anti-TNF) had lower TT measurements compared to the group taking conventional disease modifying antirheumatic drugs (DMARD) (p = 0.039).

**Conclusion:**

The thiol-disulfide balance is disturbed in favor of disulfide in SpA patients compared to healthy volunteers. Native and total thiol measurements correlate with acute phase reactants and might be used to monitor disease activity. Anti-TNF therapy might control the oxidative degenerative process better than the conventional DMARD in SpA patients.

## 1. Introduction

Seronegative spondyloarthritis (SpA) is a chronic disease that mostly presents with axial inflammation, oligoarthritis, enthesitis, and uveitis, and less commonly presents with dactylitis, erythema nodosum, and enteral involvement. Subgroups of SpA consist of ankylosing spondylitis (AS), undifferentiated spondyloarthritis (USpA), enteropathic arthritis, reactive arthritis, psoriatic arthritis, and juvenile spondyloarthritis [1].

Reactive oxygen species (ROS) are often synthesized either due to physiologic mechanisms, such as aerobic metabolism and nitric oxide (NO) synthesis, or pathologic mechanisms, such as malignancy, smoking, infections, and rheumatologic conditions [2]. Excessive ROS production that cannot be counterbalanced with redox buffer capacity may lead to cellular damage [3]. Oxidative stress is mainly controlled by thiols, also known as mercaptans, which play an essential role as a radical scavenger [4]. Thiols consist of the main compound of the antioxidation pool in human metabolism. They form reversible disulfide bridges via oxidizing ROS and are reduced to thiol groups again when conditions change in favor of antioxidants. There are several approaches for measuring oxidative stress in human metabolism, based on both direct and indirect detection methods. There is the direct measurement of free radicals, such as hydrogen peroxide, singlet oxygen, hypochlorite, and nitric oxide, and the indirect measurement of oxidative stress products, also called redox biomarkers, such as glutathione, dityrosine, 8-hydroxy-2′-deoxyguanosine (8OHdG), thiol, and disulfide [5,7]. Erel and Neselioglu developed a novel technique to determine thiol and disulfide levels with high accuracy [8].

Up to now, a number of studies have demonstrated that oxidative stress contributes to the pathogenesis of chronic degenerative diseases and rheumatologic diseases, including SpA [9–17]. So far, the clinical significance of thiol-disulfide balance in patients with SpA has been understudied. The present study aims to test the difference in serum dynamic thiol-disulfide levels using Erel’s method among SpA patients (with two subgroups of USpA and AS patients) and healthy controls. The secondary aim of the study is to compare the dynamic thiol-disulfide levels between two different treatment groups of the patients, those using antitumor necrosis factor (anti-TNF) and those using conventional disease modifying antirheumatic drugs in combination with nonsteroidal antiinflammatory drugs (DMARD+NSAID).

## 2. Materials and methods

A total of 144 patients with AS or USpA and 80 healthy volunteers were included. All of the included patients were admitted to our out-patient rheumatology department consecutively. All of the participants were over 18 years old. The diagnoses of AS and USpA were made according to the modified New York criteria and the ASAS classification criteria for axial SpA [18,19]. In addition to being divided according to their disease subtype, the patient group was also divided according to their treatment modality (anti-TNF versus DMARD+NSAID).

Patients were excluded if they had a history of malignancy, smoking, another overlapping rheumatic disease, liver or renal dysfunctions, or any other chronic diseases. The healthy control group had no known previous medical history of any chronic disease, smoking, or additional substance use. The control group was selected in an age-and-sex-matched manner regarding the features of the patient group. Demographic characteristics of the patients and healthy controls are shown in Table 1. The findings of physical examination, plain radiography of joints, and sacroiliac MR imaging (as needed) were evaluated in the patient group. The Bath ankylosing spondylitis disease activity index (BASDAI) score was used to assess disease activity [20]. The HLA-B27 gene analysis was included in the analysis. Peripheral blood samples obtained from the healthy volunteers and the patients were analyzed for native thiol (NT), total thiol (TT), and disulfide (D) levels. Concomitantly, complete blood count, erythrocyte sedimentation rate (ESR), C-reactive protein (CRP), and biochemical parameters were evaluated. Disulfide and thiol levels were measured using a novel fully automated colorimetric method developed by Erel and Neselioglu [8]. Disulfide/native thiol (D/NT), disulfide/total thiol (D/TT), and native thiol/total thiol (NT/TT) ratios were calculated. The patient and healthy control groups were compared in terms of demographic characteristics, laboratory findings, NT, TT, disulfide, and the D/NT, D/TT, and NT/TT ratios. The USpA and AS groups were also compared regarding HLA-B27 positivity, disease activity scores, NT, TT, disulfide, and the D/NT, D/TT, and NT/TT ratios.

Written informed consent was obtained from all participants. Ethical approval was obtained from our local ethics committee.

### 2.1. Statistical analysis

The Shapiro–Wilk test was used to determine whether the variables have parametric or nonparametric distribution. The descriptive statistics of the data with normal distribution were given as mean ± standard deviation. The descriptive statistics of the variables, which have a nonnormal distribution, were reported as median with data range (minimum to maximum). Student’s t-test and the Mann–Whitney U test were used for pairwise comparisons regarding parametric or nonparametric distribution of the continuous variables. Levene’s test was used for testing the homogeneity of the variances as needed.

The Kruskal–Wallis test was used to compare the age variable between patient subgroups (AS and USpA) and the control groups. A one-way analysis of variance test was applied to assess the variance analysis of the subgroups according to NT, TT, disulfide, D/NT, D/TT, and NT/TT. In addition, since the sex variable was significantly different between the groups, the NT, TT, and disulfide values and the D/NT, D/TT, and NT/TT ratios were evaluated with a one-way analysis of covariance (ANCOVA) test to eliminate the effect of sex. A post hoc Bonferroni test was used for multiple pairwise comparisons.

Due to the nonparametric distribution of the parameters, the Mann–Whitney U test was performed to compare the groups according to BASDAI, age, CRP, and ESR. The Pearson correlation and Spearman correlation tests were applied to identify any association between NT, TT, disulfide, D/NT, D/TT, and NT/TT and BASDAI, age, CRP, and ESR. To compare categorical variables between groups, the chi-square test was applied. IBM SPSS Statistics version 21.0 was used for the statistical analysis. The level of statistical significance was set at p < 0.05.

## 3. Results

A total of 144 patients and 80 healthy volunteers were included in the study. In the patient group, 67% (n = 97) had a diagnosis of USpA and 32% (n = 47) had AS. The SpA group and the healthy group were similar according to sex (p = 0.167) and age (p = 0.187) distribution. As expected, there was a male preponderance in the AS group compared to the USpA group (53% vs 17%, p < 0.001). The median age of patients in the AS group was 46 years (range 28–66), 50 years in the USpA group (range 23–66), and 46 years in the control group (range 24–72) (Table 1). There was no significant difference between the AS and USpA groups regarding disease duration, age at diagnosis, BASDAI, ESR, and CRP levels (Table 2). HLA-B27 results were available for 45.8% (n = 66) of the patients. Among the patients who had the HLA-B27 analysis, 52.9% (n = 9) of the AS group and 30.6% (n = 15) of the USpA group were found to be positive. As expected, HLA-B27 positivity was higher in the AS group, but this did not reach statistical significance (p = 0.09).

Native thiol levels were significantly lower in the SpA group than in the healthy volunteer group (p < 0.001). Total thiol levels were also lower in the SpA group but there was no statistically significant difference (p = 0.074). Disulfide levels, D/NT, and D/TT were significantly higher in the SpA group than in the healthy control group (p < 0.001). On the other hand, the NT/TT ratio was significantly lower in both SpA groups than in healthy control group (p < 0.001). Details of the comparisons between groups in terms of thiol-disulfide levels are shown in Tables 3 and 4.

NT and TT levels were negatively correlated with CRP level (*r* = −0.45, p < 0.001) and ESR (*r* = −0.396, p < 0.001 and *r* = −0.351, p < 0.001, respectively). Disulfide, D/NT, and D/TT were also negatively correlated with disease duration (*r* = −0.196, p < 0.039*; r* = −0.192, p < 0.043; and *r* = −0.192, p < 0.043, respectively). However, no correlation was found between BASDAI score and the thiol-disulfide parameters. Results of correlation analysis between the dynamic thiol-disulfide parameters and variables such as BASDAI score, disease duration, CRP, and ESR are given in Table 5.

The patient treatment modality subgroups (anti-TNF and conventional DMARD) were compared in terms of NT and TT levels. TT levels were statistically lower in patients using anti-TNF agents compared to conventional DMARD treatment (p = 0.039). The details are showed in Tables 6 and 7.

## 4. Discussion

This study found that native thiol and native thiol/total thiol ratio were significantly lower for SpA patients than the healthy control group, and disulfide, D/NT, and D/TT ratios were significantly higher in SpA patients. In addition, native and total thiol levels were lower and serum disulfide levels were higher in the USpA group compared to the AS group.

Recently, there has been a growing number of publications focused on the relationship between oxidative stress and chronic diseases such as hypertension, asthma, cardiovascular diseases, and rheumatologic diseases [21–24]. Thiols play a fundamental role in reducing oxidative stress and protecting from cellular damage by forming disulfide bridges with covalent bonds [4]. Thus, the level of thiol in the body can be an important indicator of the antioxidant capacity of metabolism. In our study, serum thiol (native and total) levels were consistently significantly lower in the SpA patients compared to the control group. Similarly, in a small group of patients, Dogru et al. showed that native thiol and total thiol levels were significantly low in AS patients. They also reported a negative correlation between BASDAI and thiol levels [22], but our study, based on a larger patient group, did not find a correlation between BASDAI and thiol levels. BASDAI score is based on a questionnaire, so it may have subjective results. This may explain the lack of correlation between BASDAI and thiol-disulfide levels. Another study conducted to evaluate thiol-disulfide balance in AS also found that total thiol levels and N/TT ratio are lower in AS patients compared to healthy controls [2]. They did not find any significant correlation between the DMARD and anti-TNF treatment groups. Conversely, our study found that the anti-TNF group had higher total thiol levels, which may indicate that anti-TNF therapy can control oxidative stress more effectively than conventional DMARD.

In this study, thiol levels were found to be significantly lower in USpA patients than in AS patients. In addition, NT and TT levels were not significantly different between the AS group and the healthy control. This may be explained by the widespread utilization of effective anti-TNF treatment modalities in recent years. Ugan et al. reported that infliximab, an anti-TNF drug, may protect against oxidative stress and apoptotic cell death in AS patients and also regulate the signal mechanisms [25].

Thiol groups compensate for reactive oxygen species (ROS) by forming disulfide bridges. Therefore, serum disulfide levels may be an indicator of metabolic oxidative stress. Accordingly, in our study, the disulfide level was significantly higher in the SpA group compared to the control group. In the subgroup analyses, the disulfide level was found to be significantly higher in the USpA group compared to the AS group. In the USpA group, lower thiol and higher disulfide levels were found. These results may support the approach of intensifying treatment with biological agents, such as anti-TNF agents, in USpA patients with active disease.

The strong negative correlation between acute phase reactants (CRP and ESR) and thiol levels may imply that thiol measurements can be used to monitor disease activity. These results seem to be consistent with those of other researchers, who found thiol levels to correlate with ESR and CRP levels [22,26].

A positive correlation between disease duration and disulfide levels may imply that oxidative stress is a complex process that is not only triggered by acute stress but may also accumulate chronically. Recent studies also support the hypothesis that thiol-disulfide counterbalance moves towards the disulfide side in the course of a chronic inflammatory disease [27–29].

Although this study has demonstrated that native and total thiol levels correlate with acute phase reactants, evaluating the variations in the thiol-disulfide levels over time could provide further information to elucidate the clinical significance of thiol-disulfide levels. This point can be considered a limitation of this study. Another possible limitation is that only active smokers were excluded from the study. Being a former smoker was not assigned as an exclusion criterion, which could be a minor confounder of the results.

In conclusion, thiol-disulfide balance is disturbed in favor of disulfide in SpAs. Native thiol measurement can also be used to monitor disease activity. Anti-TNF therapy, one of the backbone therapies for AS, may also help to control the oxidative degenerative process in SpA. To the best of our knowledge, this is the first study comparing oxidative stress in AS and USpA patients. Further studies are needed to provide new insights into disrupted thiol-disulfide homeostasis in SpA, which may become a potential therapeutic target in the future.

## Figures and Tables

**Table 1:** Comparison of sex and age distribution of the patient and control groups.

USpA n (%) (n = 97)		SpA (n = 144)		Healthy control n (%) (n = 80)	p
		AS n (%) (n = 47)			
Sex n (%)	F	80 (82.5)	22 (46.8)	50 (62.5)	0.167
	M	17 (17.5)	25 (53.1)	30 (37.5)	
Median age, years (range)		50 (2369)	46 (2866)	46 (2472)	0.273

SpA: spondyloarthritis; USpA: undifferentiated spondyloarthritis; AS: ankylosing spondylitis; F: female; M: Male.

**Table 2:** Comparison of the USpA and AS groups in terms of disease duration, diagnosis age, BASDAI score, ESR, CRP, HLA-B27, and treatment.

	UspA (n = 97)	AS (n = 47)	p
Median duration of disease, year (range)	7 (2–12)	7 (1–13)	0.315
Median age at diagnosis, year (range)	44.5 (16–61)	37.5 (21–60)	0.01
Median BASDAI (range)	5.5 (1.3–9.3)	5.6 (1.4–8.4)	0.44
Median ESR mm/h (range)	21.0 (1–111)	13 (2–54)	0.144^*^
Median CRP mg/dL (range)	4.3 (1–79)	1 (1–77)	0.16^*^
HLA-B27 n (%)	15 (30.6)	9 (52.9)	0.099

USpA: undifferentiated spondyloarthritis; AS: ankylosing spondylitis; BASDAI: Bath ankylosing spondylitis disease activity index; ESR: erythrocyte sedimentation rate; CRP: C-reactive protein; HLA-B27: human leucocyte antigen.

**Table 3:** Comparison of dynamic thiol-disulfide values between patients and healthy controls.

	USpA (n = 97) mean (SD)	AS (n = 47) mean (SD)	Healthy control (HC) (n = 80) mean (SD)	p*
NT, μmol/L	432.4 (±41.0)	455.6 (±48.1)	468.1 (±43.1)	<0.001
TT, μmol/L	476.6 (±41.1)	490.6 (±52.8)	492.3 (±41.1)	0.074
Disulfide, μmol/L	22.1 (±7.0)	17.5 (±9.2)	12.0 (±6.0)	<0.001
D/NT	5.1 (±1.8)	3.8 (±2.0)	2.6 (±1.4)	<0.001
D/TT	4.6 (±1.4)	3.5 (±1.7)	2.4 (±1.2)	<0.001
NT/TT	90.6 (±2.9)	92.9 (±3.4)	95 (±2.5)	< 0.001

* p was obtained from a one-way ANCOVA after eliminating the sex effect.

USpA: undifferentiated spondyloarthritis; AS: ankylosing spondylitis; NT: native thiol; TT: total thiol; D/NT: disulfide/native thiol; D/TT: disulfide/total thiol; NT/TT: native thiol/total thiol.

**Table 4:** Pairwise comparison of dynamic thiol-disulfide values between groups.

		USpA (n = 97)	AS (n = 47)	Healthy control (HC) (n = 80)
NT, μmol/L	USpA	NA	p = 0.021	p < 0.001
	AS	p = 0.021	NA	p = 0.304
	HC	p < 0.001	p = 0.304	NA
TT, μmol/L	USpA	NA	p = 0.244	p = 0.071
	AS	p = 0.244	NA	p = 0.978
	HC	p = 0.071	p = 0.978	NA
Disulfide, μmol/L	USpA	NA	p = 0.004	p < 0.001
	AS	p = 0.004	NA	p < 0.001
	HC	p < 0.001	p < 0.001	NA
D/NT	USpA	NA	p < 0.001	p < 0.001
	AS	p < 0.001	NA	p < 0.001
	HC	p < 0.001	p < 0.001	NA
D/TT	USpA	NA	p < 0.001	p < 0.001
	AS	p < 0.001	NA	p < 0.001
	HC	p < 0.001	p < 0.001	NA
NT/TT	USpA	NA	p < 0.001	p < 0.001
	AS	p < 0.001	NA	p < 0.001
	HC	p < 0.001	p < 0.001	NA

USpA: undifferentiated spondyloarthritis; AS: ankylosing spondylitis; NA: not applicable; NT: native thiol; TT: total thiol; D/NT: disulfide/native thiol; D/TT: disulfide/total thiol; NT/TT: native thiol/total thiol; p was obtained from a one-way ANCOVA with Bonferroni correction test for each pairwise comparison after eliminating the sex effect.

**Table 5:** The results of correlation analysis between dynamic thiol-disulfide parameters and BASDAI score, disease duration, CRP, and ESR.

		NT μmol/L	TT μmol/L	Disulfide μmol/L	D/NT %	D/TT %	NT/TT %
BASDAI	cc	−0.092	−0.077	0.113	0.142	0.142	−0.142
	p	0.374	0.458	0.274	0.166	0.166	0.166
	n	96	96	96	96	96	96
Disease duration, years	cc	0.113	0.025	*−0.196* ^*^	*−0.192* ^*^	*−0.192* ^*^	0.192^*^
	p	0.236	0.792	*0.039*	*0.043*	*0.043*	*0.043*
	n	111	111	111	111	111	111
CRP, mg/dL	cc	*−0.345* ^**^	*−0.308* ^**^	0.083	0.137	0.137	−0.137
	p	*0.000*	*0.001*	0.381	0.151	0.151	0.151
	n	112	112	112	112	112	112
ESR, mm/h	cc	*−0.396* ^**^	*−0.351* ^**^	0.086	0.164	0.164	−0.164
	p	*0.000*	*0.000*	0.371	0.085	0.085	0.085
	n	111	111	111	111	111	111

BASDAI: Bath ankylosing spondylitis disease activity index; ESR: erythrocyte sedimentation rate; CRP: C-reactive protein; cc: correlation coefficient; NT: native thiol; TT: total thiol; D/NT: disulfide/native thiol; D/TT: disulfide/total thiol; NT/TT: native thiol/total thiol.

**Table 6:** Comparison of thiol-disulfide parameters according to treatment modality in patients with SpA.

	Anti-TNF (n = 38)	DMARD+NSAID (n = 106)	p^*^
Native thiol, μmol/L mean (SD)	450 (57.1)	436 (38.5)	0.001
Total thiol, μmol/L mean (SD)	495 (56.3)	476 (39.9)	0.007
Disulfide, μmol/L mean (SD)	22.1 (9.3)	19.8 (7.5)	0.062
D/NT, % mean (SD)	5.0 (2.3)	4.6 (1.8)	0.155
D/T, % mean (SD)	4.4 (1.9)	4.1 (1.5)	0.171
NT/TT, % mean (SD)	91.0 (3.8)	91.6 (3.0)	0.171

NT: native thiol; TT: total thiol; D/NT: disulfide/native thiol; D/TT: disulfide/total thiol; NT/TT: native thiol/total thiol; SD: standard deviation; Anti-TNF: antitumor necrosis factor; DMARD: disease-modifying antirheumatic drugs; NSAID: nonsteroidal antiinflammatory drugs.

**Table 7:** Patient groups according to treatment characteristics.

Treatment		Anti-TNF n (%) (n = 38)	DMARD+NSAID n (%) (n = 106)	p
Age, years (SD)		46 (±12)	48.9 (±8.9)	0.134**
Sex	F	18 (47.4)	85 (80.2)	<0.0001*
	M	20 (52.6)	21 (19.8)	
SpA	AS	20 (52.6)	27 (25.5)	0.002*
	SpA	18 (47.4)	79 (74.5)	

USpA: undifferentiated spondyloarthritis; AS: ankylosing spondylitis; Anti-TNF: antitumor necrosis factor; DMARD: disease-modifying antirheumatic drugs; NSAID: nonsteroidal antiinflammatory drugs;

* = chi-square test;

** = Student’s t-test.
